# A Static-99R Validation Study on Individuals With Mental Disorders: 5 to 20 Years of Fixed Follow-Up After Sexual Offenses

**DOI:** 10.3389/fpsyg.2021.625996

**Published:** 2021-02-02

**Authors:** Christian Baudin, Thomas Nilsson, Joakim Sturup, Märta Wallinius, Peter Andiné

**Affiliations:** ^1^Centre for Ethics, Law and Mental Health, Department of Psychiatry and Neurochemistry, Institute of Neuroscience and Physiology, The Sahlgrenska Academy at University of Gothenburg, Gothenburg, Sweden; ^2^Department of Forensic Psychiatry, National Board of Forensic Medicine, Gothenburg, Sweden; ^3^Forensic Psychiatric Clinic, Sahlgrenska University Hospital, Gothenburg, Sweden; ^4^Swedish Police Authority, Stockholm, Sweden; ^5^Research Department, Regional Forensic Psychiatric Clinic Växjö, Växjö, Sweden; ^6^Lund Clinical Research on Externalizing and Developmental Psychopathology, Child and Adolescent Psychiatry, Department of Clinical Sciences, Lund, Lund University, Lund, Sweden

**Keywords:** Static-99R, validation, sex offender, mental disorder, risk, risk assessment, discrimination, calibration

## Abstract

‘The Static-99R is one of the most commonly used risk assessment instruments for individuals convicted of sexual offenses. It has been validated for use on many populations, but few studies specifically target and describe individuals with mental disorders. Additionally, research on the discriminative properties (how well the instrument separates recidivists from non-recidivists) of the instrument over longer follow-up periods is scarce. This article evaluated the validity of the Static-99R using a cohort of individuals with mental disorders convicted of sexual offenses in Sweden (*N* = 146) with fixed 5-year (*n* = 100), 10-year (*n* = 91), 15-year (*n* = 79), and 20-year (*n* = 36) follow-up periods. A Static-99R cut score of 6 demonstrated the highest Youden index, maximizing sensitivity (72.7%) and specificity (74.2%), with 25.8% of recidivists correctly assumed to reoffend sexually and 95.7% of non-recidivists correctly assumed not to. The Static-99R instrument demonstrated adequate discrimination (AUC = 0.79, CI 95% = 0.70–0.87, and OR = 1.45, CI 95% = 1.14–1.84, *p* < 0.001, 5-year fixed follow-up), with only marginal differences for 10-, 15-, and 20-year fixed follow-up (AUC = 0.73, 0.74, and 0.74 and OR = 1.31, 1.36, and 1.40, respectively). Calibration (quantifying risk and correspondence with the instrument’s norms) was acceptable (Brier = 0.088, P/E = 0.70, E/O = 1.43), with the routine sample norms displaying a decisively better fit to the study cohort compared to the high-risk/high-need sample norms. The results affirm the recommendation that, when in doubt and where there is no recent local norm group large enough available, the Static-99R routine sample found in the evaluators’ workbook should be used.

## Introduction

An important goal of any sex offense legal sanction is to stop the convicted person from committing further sex offenses, through incapacitation, rehabilitation, or both. According to the risk-need-responsivity model, systematic risk assessments are required as a core principle in order to offer proportionate risk-reducing treatment to the person in question (Andrews et al., 1990; Polaschek, 2012). One way of looking at structured risk assessment instruments is to categorize them into three distinct types: structured professional judgment, actuarial, and mechanical. Structured professional judgment instruments are essentially extended clinical judgments which may or may not use empirically derived risk factors. Actuarial instruments exclusively focus on empirically derived risk factors, resulting in a total score and a related probability of recidivism. Mechanical instruments resemble a combination of the previous two but tend to lack the empirical basis required and either are used as is or have not yet gathered enough data to be classified as actuarial (Helmus, 2018).

In forensic settings, the use of actuarial risk assessment instruments appears to be most prevalent (Neal and Grisso, 2014). One such instrument is the Static-99R, which is commonly used among clinicians to assess the recidivism risk for individuals convicted of sex offenses in many countries (Rice et al., 2014; Kelley et al., 2020). Its interrater reliability has been demonstrated as high, with an intraclass correlation coefficient (ICC) as high as 0.85 for experienced assessors and 0.71 for less experienced assessors (Phenix et al., 2016a). The instrument consists of ten items empirically linked to sex offense recidivism, it can be scored objectively, and it requires minimal training (Phenix et al., 2016a). Furthermore, a Static-99R assessment can be exclusively based on documentation and therefore does not require any interviews with the assessed person.

While the previous version of the instrument, called Static-99, better predicts recidivism under certain conditions (Rettenberger et al., 2013), new research focuses almost exclusively on the revised edition, which contains a number of important changes. For example, in accordance with the United States Council of State Governments Justice Center’s five proposed risk levels for general reoffending, published in 2017 (Hanson et al., 2017b), the Static-99R was restructured into five risk categories the year before (Phenix et al., 2016a). Besides advocating for a more standardized view of recidivism risk, this development also attempts to simplify the communication of risk to those with less experience of the particular details of risk assessments. In addition to adopting this modernization, the developers of the Static family of instruments strongly advise moving away from the use of the Static-99 in favor of using the Static-99R in clinical settings (Phenix et al., 2016a).

Individuals convicted of sexual offenses and diagnosed with a mental disorder are commonly referred to as “mentally disordered sexual offenders” (MDSOs)^1^. They constitute a subgroup demonstrating substantial psychiatric comorbidity, such as psychotic disorders, personality disorders, intellectual disabilities, and substance use disorders (Harris et al., 2010a; Stinson and Becker, 2011; Kingston et al., 2015) and may present differently with regard to recidivism and treatment needs. While the older version of the Static-99 has been validated using a Swedish general prison population (Sjöstedt and Långström, 2001), it is necessary to assess the applicability of the revised Static-99R by validating its use on a Swedish cohort comprising MDSOs. It is not currently known what norm group – if any – is preferable with regard to Swedish MDSOs, complicating the use of the instrument by clinicians involved in correctional treatment as well as those working with forensic psychiatric care.

Currently there are two established norm groups suggested for use with the Static-99R, known as the “routine sample” and the “high-risk/high-need sample,” together comprising samples from seven countries on two continents (Helmus et al., 2012b; Phenix et al., 2016b). The high-risk/high-need sample was constructed with particularly violent individuals and MDSOs in mind. Researchers have previously pointed out that the choice of norm group for comparisons with any offender sample greatly affects the outcome, and there is as of yet no consensus on any particular method for deciding which norm group should be used (DeClue and Zavodny, 2013; Elwood et al., 2017). Naturally, this issue is also relevant when assessing MDSOs.

Finally, most Static-99R studies focus on fixed follow-up periods of 5 years or less (Phenix et al., 2016a), but the predictive ability of the Static-99R has been found to be acceptable for follow-up periods of 10 years (Smid et al., 2014; Lee et al., 2018; Olver et al., 2018). However, these long-term fixed follow-up studies over periods of 10 years or more are rare, and results are therefore uncertain.

Therefore, the aims of this study are: i) to clarify how the Static-99R discriminates between recidivists and non-recidivists in a Swedish MDSO population, ii) to calibrate the Static-99R by assessing how well it quantifies recidivism risk and which of the available norm groups should be used when communicating risk in said population, and iii) to establish the long-term (for 5 to 20 years) predictive validity of the Static-99R for MDSOs. Continually validating any risk assessment instrument in new populations is a task both arduous and important, but new findings may be integrated into future revisions of the Static-99R norms, further bolstering the international applicability of this risk assessment instrument.

## Materials and Methods

### Legal Setting

In Sweden, individuals who commit criminal offenses and who suffer from a severe mental disorder (i.e., major mental illnesses such as psychotic syndromes, severe developmental syndromes, and severe personality disorders with either compulsive elements or psychotic episodes) may be precluded from being sentenced to prison and can instead be sentenced to compulsory forensic psychiatric care. In order for the court to decide on a suitable sanction, a pretrial forensic psychiatric investigation (FPI) is commonly undertaken, resulting in a written report. The FPIs are conducted during a 4-week period by the National Board of Forensic Medicine. A trained team comprising a forensic psychiatrist, a forensic clinical psychologist, and a forensic social worker, as well as ward staff, carry out the FPI. Any psychiatric diagnoses ascribed are specified according to the latest version of the Diagnostic and Statistical Manual classification system (American Psychiatric Association, 2013). While prison sentences most often are time-limited, sentences for forensic psychiatric care are not. Instead, an individual sentenced to forensic psychiatric care must undergo treatment, be found no longer to suffer from a severe mental disorder, and demonstrate a low risk of reoffending before being released (Svennerlind et al., 2010).

### Static-99R

The risk assessment instrument Static-99R consists of ten items, where all but items 1 and 5 are dichotomous, resulting in 0 or 1 point being added to the total score. Item 1 (“age at release from index sex offense”) is scored 1 for ages 18 to 34.9, 0 for ages 35 to 39.9, −1 for ages 40 to 59.9, and −3 for ages 60 and older. Item 2 (“ever lived with a lover for at least two years”) is scored 0 for yes and 1 for no. This is the only item that may be omitted due to lack of information, resulting in a score of 0. Item 3 (“index non-sexual violence, any convictions”) is scored 0 for no and 1 for yes. Item 4 (“prior non-sexual violence, any convictions”) is scored 0 for no and 1 for yes. Item 5 (“prior sex offenses, charges as well as convictions”) is scored 0 for no charges or no convictions, 1 for 1–2 charges or 1 conviction, 2 for 3–5 charges or 2-3 convictions, and 3 for more than 6 charges or more than 4 convictions. The highest number of charges or convictions takes precedence, meaning an individual with no charges and more than 4 convictions receives a score of 3. Item 6 (“4 or more prior sentencing dates, excluding index”) is scored 0 for 3 or less and 1 for 4 or more. Item 7 (“any convictions for non-contact sex offenses”) is scored 0 for no and 1 for yes. Item 8 (“any unrelated victims”) is scored 0 for no and 1 for yes. Item 9 (“any stranger victims”) is scored 0 for no and 1 for yes. The stranger requirements are strict and reserved for when the assessed and the victimized individual have known each other for less than 24 h without any interaction between them (Phenix et al., 2016a). Lastly, item 10 (“any male victims”) is scored 0 for no and 1 for yes. The total score, between −3 and 12, results in one out of five recommended risk levels: level I – *very low risk* (scores of −3 to −2), level II – *below average risk* (scores of −1 to 0), level III – *average risk* (scores of 1 to 3), level IVa – *above average risk* (scores of 4 to 5), and level IVb – *well above average risk* (scores of 6+).

In the present study, three of the authors scored between 54 and 66 cases each, including 15 cases used for assessing inter-rater reliability (approximately 10% of the total number of cases). The ICC was then calculated using a two-way mixed effects absolute agreement single rater model (McGraw and Wong, 1996). An ICC of 0.89 (CI 95% = 0.76-0.94, *p* < 0.001) was achieved, which is commonly interpreted as between “good” and “excellent” (Gross, 2020) – or well above “strong” (Rice and Harris, 2005). These results are in line with the Static-99R coding rules which found ICC 0.84–0.95 across 11 studies (Phenix et al., 2016a).

Swedish law differs from Canadian law, and the coding rules of the Canadian-developed Static-99R must therefore be interpreted as closely as possible to the developers’ intent. A version of the coding rules has been translated into Swedish (Harris et al., 2010b), although it does not appear to follow the recommended procedures for translating instruments by applying either back-translation or comparisons of multiple independent translations of the text (Gudmundsson, 2009). For this reason, some adaptations of the original coding rules were made for this study. In Sweden, an offense typically results in one of several types of judicial decisions, and the subjects included in the current study were affected by the following: verdicts, waivers of prosecution, and summary impositions of a fine. Verdicts are uncomplicated when using the Static-99R, but waivers and summary impositions of a fine may affect the total score as follows: In short, a waiver of prosecution may be issued for minor offenses where the subject is concurrently being prosecuted for a graver offense. A summary imposition of a fine is commonly suggested for minor offenses that may result in only a fine or a conditional sentence requiring that the person admit to the offense. Any type of judicial decision involving a sex offense counts as a prior sex offense for item 5. Any conviction resulting in a verdict, a waiver of prosecution, or the specific sanction of a suspended sentence counts as a prior sentencing date for item 6. In addition, a summary imposition of a fine counts as a suspended sentence; that is it should be noted for item 6 if the range of punishment requires an imprisonment of 14 days or longer. As such, only some of the instances where the judicial decision is a summary imposition of a fine may count with regard to item 6.

The release date, used for calculating age at release in item 1, was defined as (i) the date of release from prison – conditionally or otherwise – which in Sweden commonly occurs after two thirds of the time stated in the verdict, (ii) the date of release from compulsory forensic psychiatric care, and (iii) the sentencing date for subjects sentenced to probation, effectively resulting in a release.

For the present study, an act of recidivism was identified as a conviction for a new sex offense according to chapter six of the Swedish Penal Code. According to the coding rules, all post-index offense convictions, charges, and arrests for a new sex offense count as recidivism (Phenix et al., 2016a). In Sweden, all convictions since 1973 are kept on file and are available for research. Charges are not as readily available but are commonly appended to written court verdicts. This is unfortunately not true for all cases, however. Arrest records are not available, and this type of recidivism therefore cannot be measured and used for risk assessments with the Static-99R. Please note that any non-sexual reoffenses are ignored for the purposes of the current study and will therefore not be counted. Note also that four subjects reoffended during ongoing forensic psychiatric care. According to the coding rules, the assessed individuals are not technically able to reoffend sexually before the release date; these offenses should be converted into new index sex offenses. The new offenses occurred outside of the years of inclusion and were not accompanied by a new FPI; completely excluding the subjects would unnecessarily deplete the cohort. This is particularly problematic considering that studies using Static-99R risk assessments in MDSOs are uncommon. For these reasons, the offenses of the said four subjects were instead considered part of a cluster of the index offense (Phenix et al., 2016a). While regrettable, adaptation of the coding rules for a real life scenario is very common in validation studies (Rossegger et al., 2013).

### Study Population and Data Collection

**FIGURE 1:**
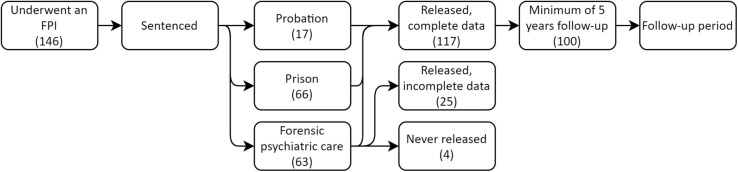
Flow chart illustrating adult males who sexually victimized a person over the age of 15 and subsequently underwent a forensic psychiatric investigation during 1993–1997.

Between January 1, 1993 and December 31, 1997, there were 857 persons convicted of a sex offense with a victimized person over the age of 15 in Sweden. Out of these offenders, 146 adult males underwent a court-ordered FPI due to suspicion of a severe mental disorder – an offender group also presented in a recent follow-up study (Baudin et al., 2020). Demographically, 62.3% (*n* = 91) were born in Sweden, 8.2% (*n* = 12) originated from other Nordic countries, 8.9% (*n* = 13) were from the rest of Europe, and 20.5% (*n* = 30) came from elsewhere in the world. As can be seen in Figure 1, the offenders were subsequently sentenced to prison, compulsory forensic psychiatric care, or probation. Data regarding all offenses committed by the subjects – pre-index, index, and post-index – were collected from the National Council for Crime Prevention’s register of persons found guilty of offenses, starting from January 1, 1973 and going up until December 31, 2016, marking the end of the follow-up period. After the follow-up period, the 146 subjects were subjected to blind assessment by three of the authors with the Static-99R based on information in the FPIs and the written court verdicts for the index offense, as well as for all previous sex offenses. Of the 146 subjects, four were not released from forensic psychiatric care during the follow-up period, and in 25 cases release dates were unavailable, resulting in exclusion from analyses. Additionally, of the 117 fully assessed subjects, 100 had at least 5 years of fixed follow-up and could therefore be used for all statistical analyses, including comparisons to the Static-99R norm groups sample.

Sex offenses were defined as all acts listed under chapter six in the Swedish Penal Code, including but not limited to rape, sexual molestation, and sexual coercion, and included attempted sex offenses. All index offense victimized persons were at least 15 years of age at the time of the offense, which is the age of sexual consent in Sweden, and a vast majority were female (97.3%).

Instances of recidivism were defined as a conviction for a new sex offense after the release date. While new charges may technically count as a new offense, this information is not available on request in Sweden. Roughly half of the samples included in the normative data for the development of the Static-99R exclusively used convictions and not charges, however (Phenix et al., 2016a).

The time that subjects were considered incapacitated was defined as time spent either in prison or under compulsory forensic psychiatric care and therefore not at large in the community, estimated as starting from the index offense conviction date up until the date of release. This does not equate to the sanction length stated in the sentence documents since (i) for subjects sentenced to prison, all were conditionally released, and (ii) there is no set endpoint for subjects sentenced to forensic psychiatric care, and a sanction length therefore cannot be formally calculated. A subject sentenced to probation would effectively not be incapacitated for any length of time, despite submitting to some form of supervisory control.

Time at risk was defined as the time during which the subject was released and at large in the community, and was measured from the index offense release date up until the first new sex offense conviction or, in cases of no recidivism, the date of death, migration from Sweden, or the end of the follow-up period.

### Statistical Analyses

The analytical methods are based on the recent – and comprehensive – validity study of a Swizz population by Gonçalves et al. (2020), and in particular the use of the P/E index and the Brier score as described below. When validating an actuarial instrument, discrimination and calibration are both important. Discrimination in risk assessment settings examines how well the scale separates recidivists from non-recidivists (e.g., differences between the risk scores of recidivists and non-recidivists), whereas calibration evaluates the correspondence between expected recidivism rates per score (available in the instrument’s norms) and observed or predicted recidivism rates in the current sample (Helmus and Babchishin, 2017). This applies to comparisons to both of the available norm groups: the routine sample as well as the high-risk/high-need sample suggested by the developers of the Static-99R.

For the purpose of discrimination, the area under the curve (AUC) of receiver operating characteristics (ROC) analyses and also logistic regressions with odds ratios (OR) are commonly recommended (Hanson et al., 2013; Helmus and Babchishin, 2017), although comparisons of the intercept (B_0_) and slope coefficient (B_1_) are sometimes used (Helmus et al., 2012a,b; Hanson et al., 2014). For recidivism studies, the AUC and the OR complement each other. The value of the AUC demonstrates the probability that a randomly selected recidivist would have a more divergent total score than a randomly selected non-recidivist, whereas the value of the OR demonstrates a change in relative risk linked with a one-point change in total score (Hanson et al., 2014). The AUC is particularly well suited for measuring the predictive accuracy of risk assessment instruments since it is not affected by low sex recidivism base rates (Hanson and Thornton, 1999; Reeves et al., 2018), although it may be influenced by the variance of the predictor (i.e., the total score range of the instrument) (Helmus et al., 2012b). The OR, in contrast, is not affected by said variance (Hanson, 2008). Additionally, it has previously been argued that commonly used guidelines such as those suggested by Rice and Harris (Rice and Harris, 2005) may overestimate the clinical implications of a large AUC value (Sjöstedt and Grann, 2002; Bengtson and Långström, 2007), and arguments against the use altogether of the AUC have been put forward (Helmus et al., 2012a,b). For these reasons, both the AUC and the OR are presented in the present study.

There is no clear consensus on what metrics are appropriate for analyzing and presenting calibration statistics, but various methods of comparing observed recidivism rates and probabilities in the form of expected recidivism rates is commonly used. While the rates for common Static-99R total scores are available in the Static-99R evaluators’ workbook, the original intercept and slope can be used to calculate more exact recidivism rates spanning the entire range of possible total scores for the Static-99R (Hanson et al., 2016; Phenix et al., 2016b). This method was used in the current study. When comparing probabilities and recidivism rates – or by extension the number of recidivists – variations of the E/O index as well as the Brier score have previously been suggested (Hanson, 2017; Helmus and Babchishin, 2017; Gonçalves et al., 2020). The E/O index, when used for evaluating risk assessment instruments, is the ratio of the expected number of recidivists (E) – derived from a logistic regression using the routine sample – to the observed number of recidivists in the study sample (O). With perfect calibration, the E/O index produces a value of 1, whereas a value higher or lower than 1 indicates the number of expected recidivists that exceeds or falls short of the number of observed recidivists, meaning the instrument has either overpredicted or underpredicted the recidivism (Hanson, 2017; Helmus and Babchishin, 2017). The confidence interval (CI) for the E/O index is commonly calculated using the Poisson variance for the logarithm of the observed number of recidivists from the current data and the expected number of recidivists from the Static-99R norm group most befitting the study cohort. A CI containing the value 1 is considered non-significant, meaning the expected recidivism rates are not statistically significantly different from the observed recidivism rates (Hanson, 2017; Helmus and Babchishin, 2017; Gonçalves et al., 2020). To clarify, a non-significant E/O CI result is a desirable outcome in the present study since it indicates that whichever norm group the observed recidivism rates originate from is comparable to that of the study cohort.

Gonçalves et al. (2020) suggested that a slight alteration to the E/O index may be preferable for calibration. The authors noted that ratios between the outcome of a logistic regression and an actual observed outcome, such as the E/O index, produce less comparable results than those between two logistic regressions. Instead, they suggested using solely the recidivism rates predicted by logistic regressions for the study sample (naming this variable P) as well as the chosen norm group (E), calling this the P/E index. This also eliminates the fairly common issue of being forced to divide by zero for uncommon events such as can be found in recidivism research (Hanson, 2017). We hold that both approaches are valuable to validation studies and therefore present both indexes: The E/O index is used for easier comparison with other studies, and the P/E index for practicality.

The Brier score is a measurement for comparing the predictive accuracy of two or more models with binary outcomes, such as logistic regression models (Rufibach, 2010). While it is a new tool in the field of risk assessment, the measurement is common in the fields of medicine and meteorology. For risk assessment instruments like the Static-99R, the Brier score calculates the difference between the probability of recidivism and the observed outcome of a new sex offense, for every single case in the cohort, and this is then presented as an average squared difference of all cases (Gonçalves et al., 2020). The resulting score is used for assessing how accurately the predicted probabilities of the logistic regression model fit the actual cohort data – where 0 indicates a perfect fit and 0.25 is indicative for random chance – as well as for comparing the performance of two or more logistic regression models (Rufibach, 2010). For more detail on the Brier score and its use in calibrating risk assessment instruments, please see the study by Gonçalves et al. (2020).

The Youden index is described by Gross (2020) as the cut score at which “there is a maximal difference between the true positive rate and the false positive rate – the difference between sensitivity and 1-specificity” (p. 521). The highest value indicates the greatest specificity and sensitivity of a given test, which in turn affects the post-test probabilities (Gross, 2020).

Stata/SE 16.1 (StataCorp, 2019) was used for calculating the ICC, as well as the CI of 95% for the AUC and Brier score. Additionally, jamovi 1.2.9 (The jamovi project, 2020) was used for calculating the descriptive data, logistic regressions, ROC analyses, sensitivity, specificity, positive predictive value (PPV), negative predictive value (NPV), accuracy (correctly classified cases), and Youden index. Significant results were defined as two-tailed *p*-values of *p* < 0.05, and no multiple test adjustments were used. In accordance with Feise (2002) and Perneger (1998), the clinical relevancy of the general null hypothesis (i.e., that all null hypotheses were true simultaneously) was not considered applicable for the current study. Regarding the CI, the commonly used CI of 95% was consistently calculated for all relevant analyses, despite a recent suggestion that this may be “overly conservative for forensic practice” (Elwood, 2018).

### Ethics

The Regional Ethical Review Board at the University of Gothenburg approved the study (377-17, T1056-17). Additionally, the board agreed that contacting every subject regarding sex offenses committed in the mid-90s could cause more psychological and social harm than what is ethically reasonable. As such, informed consent was not practiced with regard to the offending subjects.

## Results

### Cohort Characteristics

Table 1 provides the descriptive characteristics of the study cohort. Of the 146 subjects, 117 were fully assessed using the Static-99R since there was a lack of data on item 1 for 29 of the subjects – all of whom had been sentenced to forensic psychiatric care for their respective index offense. Four of these subjects had not been released from forensic psychiatric care at the end of the follow-up period and therefore had no release date on which to base the calculation of their age at release. For the remaining 25 patients, no date of release from forensic psychiatric care was obtained due to the inability of the respective forensic psychiatric unit to find the data in their records. Unfortunately, an additional 17 subjects participated for less than 5 years after release. This means a minimum fixed 5-year follow-up was unattainable for the calibration analyses – a recommended general practice for validation studies and a requirement reflected in the recidivism tables of the Static-99R evaluators’ workbook (Rossegger et al., 2013; Phenix et al., 2016b). This said, Table 1 demonstrates only minor differences between subjects included (*n* = 100) and subjects excluded (*n* = 46), the two exceptions being large differences in prevalence rates of psychotic disorders and, secondly, whether or not a subject was sanctioned to compulsory forensic psychiatric care as a result of the index offense. Roughly one in ten was diagnosed with intellectual disability, although this has previously been found to not adversely affect the usage of the Static-99R (Stephens et al., 2018).

**TABLE 1:** Index offense descriptive data on all subjects (*N* = 146), illustrating any differences between subjects with complete data and at least 5 years of follow-up (*n* = 100), and a column comprising subjects with incomplete data (*n* = 29) or less than 5 years of follow-up (*n* = 17).

	Subjects		
	Total cohort *N* (% of *N*)	Complete data and at least 5 years of follow-up *n* (% of *n*)	Incomplete data or less than 5 years of follow-up *n* (% of *n*)
Number of subjects	146 (100)	100 (100)	46 (100)
Pre-index sex offense	49 (33.6)	38 (38)	11 (23.9)
Psychotic disorder	38 (26.0)	18 (18)	20 (43.5)
Personality disorder	102 (69.9)	65 (65)	25 (54.3)
Intellectual disability	13 (8.9)	10 (10)	3 (6.5)
Substance use disorder	55 (37.7)	41 (41)	14 (30.4)
Sanction type			
Prison sentence	66 (45.2)	55 (55)	11 (23.9)
Forensic psychiatric care	63 (43.2)	28 (28)	35 (76.1)
Probation	17 (11.6)	17 (17)	0 (0)
Recidivism before release	4 (2.7)	4 (4)	1 (2.2)
Male victimized person	3 (2.1)	3 (3)	0 (0)
Contact offense	123 (84.2)	87 (87)	36 (78.3)
Steady partner	51 (34.9)	37 (37)	14 (30.4)
Secondary school diploma	43 (29.5)	30 (30)	13 (28.3)
Employment or studies	46 (31.5)	36 (36)	10 (21.7)

	***Mdn* (range, *SD*)**	***Mdn* (range, *SD*)**	***Mdn* (range, *SD*)**

Age at index	34.5 (17.7–71.6, 10.5)	34.2 (18.4–70.7, 10.2)	35.3 (17.7–71.6, 11.2)
Age at first conviction	21.7 (15.2–66.6, 10.1)	20.9 (15.4–66.6, 9.7)	22.8 (15.2–59.4, 11.1)
Charges convicted for	1 (1–3, 0.4)	1 (1–3, 0.4)	1 (1–3, 0.4)
Number of diagnoses	2 (0–6, 1.2)	2 (0–6, 1.2)	2 (1–6, 1.2)
Static-99R total score	4 (−2–11, 2.7)	4 (−2–11, 2.9)	4 (−1–10, 2.3)

*Only subjects with complete data and at least 5 years of follow-up were included in the discrimination, calibration, and long-term risk prediction analyses. Data for item 1 was incomplete for some subjects, either because the subjects concerned were still undergoing forensic psychiatric care at the time of the study, or there were no release dates available. Naturally, this affects the total Static-99R score row of the table (missing item 1 scored as 0 for this comparison only).*

For the 100 fully assessed subjects with at least 5 years of follow-up, the average age at release for the index offense was 37.5 years (*Mdn* = 36.4, *SD* = 10.5, range = 19.8–70.7). Those sentenced to prison (*n* = 55) had an average prison sentence length of 3.1 years (*Mdn* = 2.9, *SD* = 1.7, range = 0.3–8.0), of which the average time spent incarcerated was 2.0 years (*Mdn* = 1.8, *SD* = 1.1, range = 0.2–5.7). In comparison, subjects sentenced to compulsory forensic psychiatric care (*n* = 28) were generally incapacitated for a longer period of time, averaging 3.7 years (*Mdn* = 2.8, *SD* = 3.5, range = 0–14.3), while subjects sentenced to probation were considered to be released immediately after the verdict. In contrast to time incapacitated, the time at risk was far greater for the cohort: an average of 15.3 years (*Mdn* = 17.6, *SD* = 6.5, range = 0.3–23.5, *n* = 100), with only slight differences between those sentenced to prison (*M* = 15.3, *Mdn* = 17.6, *SD* = 6.7, range = 0.8–22.8, *n* = 55), forensic psychiatric care (*M* = 15.6, *Mdn* = 16.6, *SD* = 4.8, range = 2.1–21.9, *n* = 28), and probation (*M* = 14.6, *Mdn* = 19.9, *SD* = 8.6, range = 0.3–23.5, *n* = 17).

The average total Static-99R score for subjects with a fixed 5-year follow-up was 4.2 (*Mdn* = 4.00, *SD* = 2.9, range = −2–11, *n* = 100) and the most common risk level was III – *average risk* (34% of the subjects), followed by level IVb – *well above average risk* (31%), level IVa – *above average risk* (28%), and, to a much lesser extent, level II – *below average risk* (6%), and level I – *very low risk* (1%). The average number of years until the first new sex offense after release from the index offense was 5.3 (*Mdn* = 2.4, *SD* = 4.8, range = 0.3–14.2), where no new subjects reoffended after the 15-year follow-up mark.

### Discrimination

In short, the Static-99R predicted sexual recidivism with statistically significant accuracy for subjects with a fixed 5-year follow-up with a large effect size (AUC = 0.79, CI 95% = 0.70–0.87) in the study cohort (*n* = 100), where every unit of increase in the Static-99R total score correspondingly increased the odds of reoffending by 45% (OR = 1.45, CI 95% = 1.14–1.84, *p* < 0.001).

As seen in Table 2, the cut-off for optimal sensitivity and specificity over the first 5 years after release was a total score of 6 on the Static-99R, measured as the largest Youden index (*J* = 0.4688), although the second largest was only marginally smaller (score 4 at *J* = 0.4607). If classifying subjects with a score of less than 6 as non-recidivists and subjects with a score of 6 or more as recidivists, the Static-99R correctly classified 72.7% of reoffending subjects as recidivists (sensitivity) and 74.2% of non-reoffending subjects as non-recidivists (specificity). In total, 74% of subjects scoring 6 or higher were correctly classified (accuracy of 0.74), with 25.8% of recidivists correctly assumed to reoffend sexually (PPV) and 95.7% of non-recidivists correctly assumed not to (NPV).

**TABLE 2:** Sensitivity, specificity, positive predictive value (PPV), negative predictive value (NPV), and number of cases correctly classified (accuracy) for each Static-99R score for a 5-year fixed follow-up for individuals with mental disorders convicted of sexual offenses (*n* = 100).

Score	Sensitivity	Specificity	PPV	NPV	Accuracy
≥−2	100%	0%	11%	–	0.11
≥−1	100%	1.12%	11.11%	100%	0.12
≥0	100%	3.37%	11.34%	100%	0.14
≥1	100%	7.87%	11.83%	100%	0.18
≥2	100%	20.22%	13.41%	100%	0.29
≥3	100%	37.08%	16.42%	100%	0.44
≥4	100%	46.07%	18.64%	100%	0.52
≥5	72.73%	60.67%	18.6%	94.74%	0.62
**≥6**	**72.73%**	**74.16%**	**25.81%**	**95.65%**	**0.74**
≥7	45.45%	84.27%	26.32%	92.59%	0.80
≥8	36.36%	91.01%	33.33%	92.05%	0.85
≥9	27.27%	94.38%	37.5%	91.3%	0.87
≥10	18.18%	95.51%	33.33%	90.43%	0.87
≥11	9.09%	97.75%	33.33%	89.69%	0.88

*The line in boldface indicates the score with the highest median Youden index, although the differences between score 4 and score 6 was quite small (0.4688 compared to 0.4607).*

Considering the scoring and number of response categories of item 1 is the only change in the Static tally sheets between the two versions, the discriminative properties of the Static-99 and Static-99R were compared. No statistically significant difference was found between the two versions of the instrument (AUC = 0.80 versus 0.79, *p* = 0.326), and the differences in Youden index were small, although favoring a total score cut-off of 4 compared to 6 for the Static-99 (*J* = 0.494 versus 0.469).

### Calibration

The Brier score for the study cohort (*n* = 100) was 0.088 (observed recidivism compared to predicted recidivism), 0.092 for the routine sample (observed recidivism compared to expected recidivism), and 0.096 for the high-risk/high-need sample (again, observed recidivism compared to expected recidivism).

**FIGURE 2:**
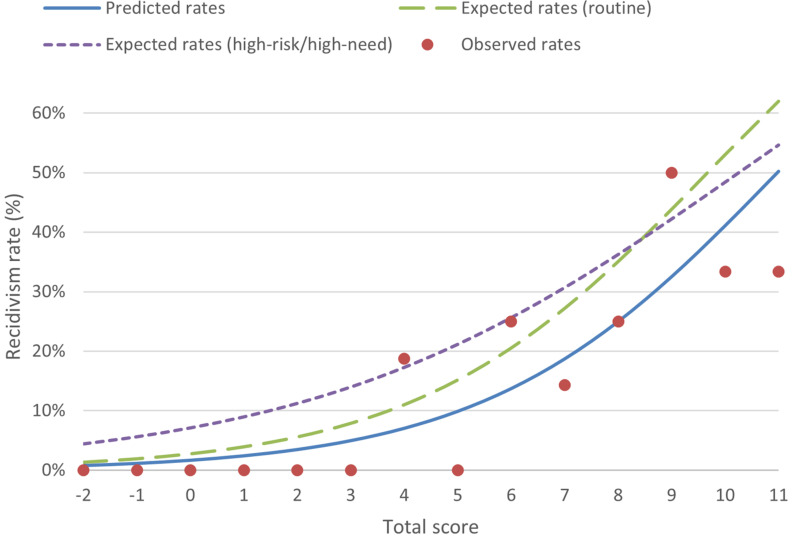
Predicted, expected (from routine sample), expected (from high-risk/high-need sample), and observed sexual recidivism rates the first 5 years after release at each Static-99R score.

A calibration plot (Figure 2) visualizes the first 5 years after release in terms of predicted and observed recidivism rates for the study cohort (*n* = 100), as well as the expected recidivism rates calculated using the norms from the routine sample as well as from the high-risk/high-need sample. Predicted recidivism rates for the study cohort were consistently lower than the expected rates derived from the routine and high-risk/high-need samples.

The trend available in Figure 2 is confirmed by the data presented in Table 3. The number of recidivists predicted over the first 5 years was 30% lower than expected when compared to the routine sample (P/E = 0.70), with marginally better prediction for recidivists categorized as risk level IVb (27% lower) compared to all lower risk levels (35–38%). No statistically significant differences were found between predicted and expected numbers of recidivists. As with the Brier scores, the total P/E values for the high-risk/high-need sample (0.54, CI 95% = 0.30–0.98) were not as good as those for the routine sample, and were statistically significant, indicating that comparisons with the routine sample were preferable, although the differences were smaller for recidivists categorized as risk level IVb (31% compared to 27%).

**TABLE 3:** Number of recidivists across Static-99R risk levels for a 5-year fixed follow-up for both routine sample and high-risk/high-need sample.

Risk levels and sample		Number of recidivists				
Routine	*N*	Observed	Predicted	Expected	E/O (CI 95%)	P/E (CI 95%)
I	1	0	0.01	0.01	–	0.62
II	6	0	0.09	0.15	–	0.62
III	34	0	1.20	1.90	–	0.63 (0.11–3.78)
IVa	28	3	2.32	3.58	1.19 (0.42–3.36)	0.65 (0.18–2.35)
IVb	31	8	7.35	10.10	1.26 (0.68–2.34)	0.73 (0.35–1.50)
Total	100	11	10.98	15.75	1.43 (0.87–2.35)	0.70 (0.39–1.26)

**High-risk/high-need**	***N***	**Observed**	**Predicted**	**Expected**	**E/O (CI 95%)**	**P/E (CI 95%)**

I	1	0	0.01	0.04	–	0.19
II	6	0	0.09	0.40	–	0.23
III	34	0	1.20	3.80	–	0.32 (0.05–1.89)
IVa	28	3	2.32	5.31	1.77 (0.76–4.15)	0.44 (0.12–1.58)
IVb	31	8	7.35	10.62	1.33 (0.73–2.42)	0.69 (0.34–1.43)
Total	100	11	10.98	20.18	1.83 (1.18–2.84)	0.54 (0.30–0.98)

*The observed number of recidivists (O) is based on the outcome for the study cohort, the predicted number of recidivists (P) in the logistic regression model for the study cohort, and the expected number of recidivists (E) in the logistic regression model for the Static-99R routine sample and high-risk/high-need sample norms for a 5-year fixed follow-up. A CI of 95% that includes 1 indicates no statistically significant difference between the predicted and the expected number of recidivists (*p* > 0.05). Due to the low number of expected recidivists, the P/E index CI could not be calculated for risk levels I and II. Additionally, division by zero made E/O indexes for risk levels I to III impossible to calculate.*

### Long-Term Predictive Validity

As seen in Table 4, the logistic regression models for recidivism over 10, 15, and 20 years all produced significant results, indicating that the Static-99R accurately predicted recidivism beyond the usual 5-year follow-up post-index offense release. Effect sizes for all follow-up periods differed only marginally when measured as AUC (0.73–0.79) and OR (1.31–1.45, CI 95% = 1.03–1.91) and may be considered large (Rice and Harris, 2005). The subject drop-out rate was quite small for the first 15 years after release (21%), with a major increase during the last 5 years (64%), but with only minor changes in the Static-99R total scores for those still in the study.

**TABLE 4:** Results of Static-99R score, logistic regression models, and effect sizes for 5 to 20 years of fixed follow-up.

	Static-99R score		Logistic regression/ROC				
Time after release	*Mdn* (*SD*, range)	*n* (% of N)	*p*	B_0_ (SE)	B_1_ (SE)	OR (CI 95%)	AUC (CI 95%)
5 years	4.19 (2.85, −2–11)	100 (100)	<0.001	−4.05 (0.85)	0.37 (0.12)	1.45 (1.14–1.84)	0.790 (0.700–0.865)
10 years	4.13 (2.86, −2–11)	91 (91)	0.015	−3.12 (0.69)	0.27 (0.11)	1.31 (1.05–1.63)	0.734 (0.633–0.823)
15 years	4.42 (2.77, −1–11)	79 (79)	0.005	−2.84 (0.68)	0.31 (0.11)	1.36 (1.10–1.69)	0.741 (0.636–0.838)
20 years	4.31 (2.67, 1–11)	36 (36)	0.030	−2.72 (0.90)	0.34 (0.16)	1.40 (1.03–1.91)	0.739 (0.578–0.879)

*The *p*-value is connected to the logistic regression.*

## Discussion

We have presented a validity study of the Static-99R assessing MDSOs in Sweden, where 146 subjects underwent an FPI and were convicted for sex offenses between the years of 1993 and 1997. Out of these subjects, 100 were fully assessed and followed up for at least 5 years and up to 20 years post-index offense release, i.e., with a fixed follow-up of 5 to 20 years. The Static-99R was found to adequately discriminate between recidivists and non-recidivists regardless of length of follow-up and to slightly (but non-significantly) underpredict the number of recidivists compared to what was expected from the norm groups. Furthermore, we found that the use of the routine sample norms fit the current study sample better with regard to the P/E index and E/O index, as well as the Brier score, as compared to the high-risk/high-need sample. These findings are in agreement with the general recommendation to use the routine sample for comparisons when no recent local norm group is available that is large enough (Hanson et al., 2013). Hanson et al. recommend at least 1,000 cases with 100 recidivists for stable logistic regression estimates assuming an overall base rate of 10% (Hanson et al., 2016). While this is an ambitious goal, it was well beyond the scope of this study to attempt to create an entirely new norm group for Swedish MDSOs.

As noted in the methods section, four subjects were reconvicted for a new sex offense before their formal release from forensic psychiatric care, three of whom had at least 5 years of follow-up after release. These new convictions were not included, in order to properly adhere to the Static-99R definition of release (Phenix et al., 2016a). Naturally, a new sex offense committed by a patient currently undergoing forensic psychiatric care would be regarded as recidivism from a clinical perspective. However, this raises questions regarding the definition of time at risk and whether a convicted person ever truly can be considered incapacitated with regard to recidivism risk.

### Discrimination – Differentiating Between Recidivists and Non-recidivists

Our findings are very similar to those presented in a recent meta-study which showed an OR of 1.45 for 8,805 subjects across 21 studies, using the routine sample (Hanson et al., 2016), and comparable, if slightly lower than, those of Gonçalves et al. (2020), which showed an OR of 1.82 and an AUC of 0.81. Using the Youden index, the optimal cut score was, by an exceedingly small margin, 6 points. At this cut score, 25.8% of high-scorers (≥6) would reoffend (PPV), whereas 95.7% of low-scorers (<6) would not (NPV). This is higher than that of Gonçalves et al. (2020), who arrived at a cut score of 4, which separates “the typical offender in the middle of the risk distribution” from those demonstrating “perceptibly higher risk” and above, according to the Static-99R coding rules (p. 11, Harris et al., 2016). However, considering the minute difference in the Youden index between the cut scores of 6 and 4, this should not be over-interpreted. In fact, the corresponding PPV and NPV values for a cut score of 4 were 18.6 and 100%, meaning all subjects in the study cohort classified as non-recidivist using this cut score managed to desist from committing new sex offenses for at least 5 years after the release date. One should not forget that a low PPV and high NPV are endemic to the field of risk assessment, however, since the base rates are extremely low and with no known false positive rate, which is why these results were expected.

### Calibration and Norm Group Comparisons

The Brier score of 0.088 demonstrated that the routine sample was a better fit compared to the high-risk/high-need sample (0.092 compared to 0.096). This is further supported by the absence of statistically significant differences for the P/E or E/O indexes across all risk categories when using the routine sample, which was not the case for the high-risk/high-need sample. The number of recidivists predicted was lower than expected when compared to Gonçalves et al. (2020) (30% lower for the total cohort compared to 4% lower found by Gonçalves et al., 2020). Generally speaking, however, the calibration findings reflect the findings of Gonçalves et al., 2020, and both studies affirm the recommendation that, when in doubt and where no recent local norm group large enough is available (Hanson et al., 2013), the Static-99R routine sample found in the evaluators’ workbook (Phenix et al., 2016b) should be used. The pattern of consistently lower recidivism rates for the study cohort shown by the calibration plot (Figure 2) was understandable considering that the slope coefficient (B_1_) was almost identical to that of the routine sample logistic regression model (0.369 versus 0.368). The plot indicates that while the routine sample exhibits a better fit, offenders still tend to reoffend at a lower rate than one would expect from their total Static-99R score.

### Long-Term Predictive Validity – 10 to 20 Years

The discriminative properties of the Static-99R also proved acceptable for follow-up periods of up to and including 20 years for the study cohort (Table 4), with only small changes over time measured as either an AUC or an OR. Compared to the few previous studies assessing the Static-99R’s predictive accuracy over 10 years of fixed follow-up, the present results were practically identical measured as the AUC: 0.73 (CI 95% = 0.63–0.82) versus 0.72 (CI 95% = 0.68–0.76) (Olver et al., 2018), 0.74 (CI 95% = 0.68–0.79) (Smid et al., 2014), and 0.75 (CI 95% = 0.65–0.85) (Lee et al., 2018). Additionally, the results exceeded that of another study using ragged (i.e., variable) follow-up and an average of 9.6 years (*SD* = 3.1) of follow-up, which found an AUC of 0.69 with no CI listed (Lehmann et al., 2013). To our knowledge, no other studies exist that present results for follow-up periods of 15 or 20 years, so further comparisons were impossible to make.

### MDSOs and the Use of the Static-99R

Despite the small differences presented in Table 1, the unfortunate exclusion of many subjects diagnosed with psychotic disorders and subjects sentenced to compulsory forensic psychiatric care inadvertently reduced the prevalence of major mental illness, which in part may be the reason the routine sample norms were a better fit. Nevertheless, the subjects included still suffered from a median number of two axis I and axis II diagnoses, including a higher prevalence rate of both intellectual disability and severe personality disorder, than those excluded. In other words, the subjects included could indeed be considered MDSOs. That said, while risk level II usually constitutes 25 to 30% of the assessed (Hanson et al., 2017a), this figure was dwarfed by the number of subjects assigned risk levels III, IVb, and IVa, despite predicted recidivism rates being lower by comparison. This was particularly distinct with regard to risk level IVb, which commonly constitutes 8% of the assessed compared to our 31% (Hanson et al., 2017a). However, our subjects’ risk levels fit the typical person convicted for a sex offense (III), as well as individuals with decidedly higher risk levels (IVa and IVb) and broad criminogenic needs (Phenix et al., 2016b), and the prevalence rates were comparable to those of Gonçalves et al. (2020).

Does this finding mean that the study cohort and, by extension, MDSOs in Sweden demonstrate a less serious risk profile? Not necessarily. For example, a large Swedish study (*N* = 8,495) found that persons convicted for their first sex offense were six times more likely to have a history of psychiatric hospitalization and three to five times more likely to have a severe mental illness when compared to the general population (Fazel et al., 2007). Similarly, Lee and Hanson (2016) found that a history of psychiatric hospitalization positively affected the recidivism risk, although the increase disappeared when controlling for other, more well-established, risk factors. The Static-99R coding rules highlight the issue by stating that the instrument shows good discrimination for MDSOs (AUC = 0.75–0.76), which is only marginally lower than the findings in the current study (AUC = 0.79) (Kelley and Thornton, 2015; Phenix et al., 2016a). In a recent literature review referenced in the Static-99R coding rules, Kelley and Thornton (2015) presented the limited data available on the prevalence of psychiatric diagnoses for both the routine sample and the regular high-risk/high-need sample. Most studies that use these samples either report nothing or describe the subjects’ clinical status in general terms (Table 1, p. 264), and the authors have suggested that the applicability of the instrument to MDSOs may therefore be questionable. This suggestion has not been affected by the addition of two supplemental studies in the updated Static-99R norms presented after publication of the review, since neither study described the prevalence of any kind of mental disorder among its subjects (Lehmann et al., 2013; Hanson et al., 2014). Despite the limited data, Kelley and Thornton (2015) concluded that the Static family of instruments – of which the Static-99R is a major player – has acceptable predictive validity for MDSO populations. In a plea for more foundational research, Kelley and Thornton (2015) ended their literature review with recommendations for future studies to report diagnoses when possible to aid in the eventual development of norms specifically targeting this very population. While we have done so in the current study, the small cohort size has prevented detailed comparisons of psychiatrically separate subgroups based on diagnoses.

### Limitations

As mentioned previously, certain offenses result in waivers of prosecution or summary impositions of a fine. These convictions never result in a written sentence document, frustrating our attempts to differentiate new sex offenses from pseudo-recidivism in some cases. Fortunately, this is a minor limitation since most sex offenses are deemed serious enough to lead to a regular verdict with a detailed sentence document, all of which was available for requisition.

Several limitations arose regarding subjects undergoing forensic psychiatric care. Firstly, 25 of these subjects were excluded as a direct result of not having a release date from the index offense sanction. Since the Static-99R coding rules strictly prohibit data from being missing for any item other than item 2, this reduced the number of subjects available for complete assessment with the instrument. Due to the length of time that had elapsed since the index offense, the transfer of some of these subjects between various care facilities was impossible to follow. Regretfully, we found no way of remedying this situation. Additionally, it is uncertain at what exact date subjects undergoing inpatient forensic psychiatric care were changed to outpatient care. Since this separates non-released subjects from released subjects, it too may advance the release date for any subject undergoing forensic psychiatric care.

The Swedish National Council for Crime Prevention has no data available on criminal offenses prior to the year 1973, which affects all studies using these datasets. For the current study, 47 of the 146 subjects (39 of the 100 included in the analyses) were 15 years old or more, which is the age of criminal responsibility in Sweden, before 1973. The number of years during which these 39 subjects could hypothetically commit offenses ranged from 1 to 33 (*Mdn* = 7). However, any additional data from before 1973 would only further increase the Static-99R total score – not reduce it, which means that the cohort would be assigned an even higher risk level than now, despite the comparatively low recidivism rates. This would only put additional weight behind the current findings.

Lastly, a common limitation for studies on sex recidivism is the reliance on official data which are assumed to underestimate “true” recidivism rates by disregarding individuals not brought to justice (Hanson and Morton-Bourgon, 2009; Scurich and John, 2019). We find no reason to believe that the current study is exempt from this issue.

### Conclusion and Implications

This study validated the use of the Static-99R on a MDSO population in Sweden. All measures (Brier score, P/E index, and E/O index) suggested that the Static-99R may be used on this subgroup and that the routine sample norm group is preferable to high-risk/high-need sample norm groups. Additionally, the Static-99R was found to significantly predict recidivism up to 20 years post-index offense release. In order to further differentiate clinical subgroups among MDSOs, future research should focus on larger national samples with 5 years of follow-up. Furthermore, any jurisdiction may benefit substantially from having an anonymized national database of Static-99R risk assessments available to researchers and clinicians as is the case in, for example, Texas, United States (Rice, 2016; Boccaccini et al., 2017). This would have the added bonus of allowing for the development of up-to-date Swedish norms, further improving the foundation on which both research and clinical practice regarding individuals convicted of sex offenses in Sweden depend.

## Data Availability Statement

The raw data supporting the conclusions of this article will be made available by the authors, without undue reservation.

## Ethics Statement

The studies involving human participants were reviewed and approved by the Regional Ethical Review Board at the University of Gothenburg. Written informed consent for participation was not required for this study in accordance with the national legislation and the institutional requirements.

## Author Contributions

TN, PA, and CB: study design and conception. TN and CB: statistical analysis. TN, PA, CB, MW, and JS: writing and review of manuscript. All authors contributed to the article and approved the submitted version.

## Conflict of Interest

The authors declare that the research was conducted in the absence of any commercial or financial relationships that could be construed as a potential conflict of interest.

## Footnotes

1 The authors have made effort to use non-stigmatizing language when possible. As such, “‘offenders” is substituted with “subjects,” “persons convicted of an offense,” “the assessed,” and so on. Likewise, “victims” is substituted with “victimized persons” or similar. However, to avoid confusing the reader with overly long sentences on some occasions, the initialism MDSO (for the commonly used field-specific phrase “mentally disordered sexual offender”) is still used, as is the less stigmatizing description “subject.”
